# *Artemisia dracunculus* L. Ethanolic Extract and an Isolated Component, DMC2, Ameliorate Inflammatory Signaling in Pancreatic β-Cells via Inhibition of p38 MAPK

**DOI:** 10.3390/biom12050708

**Published:** 2022-05-15

**Authors:** Peter Smoak, Susan J. Burke, Thomas M. Martin, Heidi M. Batdorf, Z. Elizabeth Floyd, J. Jason Collier

**Affiliations:** 1 Laboratory of Islet Biology and Inflammation, Pennington Biomedical Research Center, Baton Rouge, LA 70808, USA; peter.smoak@pbrc.edu (P.S.); thomas.martin@pbrc.edu (T.M.M.); heidi.batdorf@pbrc.edu (H.M.B.); 2 Laboratory of Immunogenetics, Pennington Biomedical Research Center, Baton Rouge, LA 70808, USA; susan.burke@pbrc.edu; 3 Laboratory of Ubitquitin Biology, Pennington Biomedical Research Center, Baton Rouge, LA 70808, USA; elizabeth.floyd@pbrc.edu

**Keywords:** botanical, inflammation, islet, cytokine

## Abstract

Non-resolving pancreatic islet inflammation is widely viewed as a contributor to decreases in β-cell mass and function that occur in both Type 1 and Type 2 diabetes. Therefore, strategies aimed at reducing or eliminating pathological inflammation would be useful to protect islet β-cells. Herein, we described the use of 2′,4′-dihydroxy-4-methoxydihydrochalcone (DMC2), a bioactive molecule isolated from an ethanolic extract of *Artemisia dracunculus* L., as a novel anti-inflammatory agent. The ethanolic extract, termed PMI 5011, reduced IL-1β-mediated NF-κB activity. DMC2 retained this ability, indicating this compound as the likely source of anti-inflammatory activity within the overall PMI 5011 extract. We further examined NF-κB activity using promoter-luciferase reporter constructs, Western blots, mRNA abundance, and protein secretion. Specifically, we found that PMI 5011 and DMC2 each reduced the ability of IL-1β to promote increases in the expression of the *Ccl2* and *Ccl20* genes. These genes encode proteins that promote immune cell recruitment and are secreted by β-cells in response to IL-1β. Phosphorylation of IκBα and the p65 subunit of NF-κB were not reduced by either PMI 5011 or DMC2; however, phosphorylation of p38 MAPK was blunted in the presence of DMC2. Finally, we observed that while PMI 5011 impaired glucose-stimulated insulin secretion, insulin output was preserved in the presence of DMC2. In conclusion, PMI 5011 and DMC2 reduced inflammation, but only DMC2 did so with the preservation of glucose-stimulated insulin secretion.

## 1. Introduction

Inflammation is a component of a variety of autoimmune and obesity-related diseases [1,2,3,4], including Type 1 (T1D) and Type 2 diabetes (T2D). While physiological inflammation is important to control infection and promote tissue repair, chronic unresolved inflammation often leads to pathological outcomes and thus frequently requires pharmacological intervention [5]. One of the major signaling pathways controlling inflammation and inflammation-related outcomes is NF-κB [6].

The NF-κB pathway can be activated by extracellular ligands [7], such as interleukin-1 (IL-1) and tumor necrosis factor (TNF), but also by intracellular signals, such as those activating the intracellular pattern recognition receptors [8]. Signals linked to the NF-κB pathway activate the inhibitor of κB kinases (IKKs), which phosphorylate the inhibitor of kB (IκB) proteins [9]. The IκB proteins hold transcriptional subunits, such as RelA/p65, within the cytoplasm until a signal is received that leads to phosphorylation by IKKs [10]. Phosphorylation of IκB proteins promotes their ubiquitination and subsequent degradation [11].

Degradation of IκB leads to p65 translocation into the nucleus where it binds to genomic κB response elements within target genes. Many such target genes encode proteins that are involved directly and indirectly in the control of inflammation (e.g., cytokines, chemokines, prostaglandin synthesis, etc.; refs. [3,12]). Indeed, many signals that ultimately trigger an inflammatory response converge on the activation of the p65 transcriptional subunit. Thus, treating unresolved inflammation frequently requires interventions that disrupt signaling through the IKKs, p38 mitogen-activated protein kinase (MAPK), and reduce NF-κB p65 transcriptional activity [13,14,15]. This approach is typically used to treat a wide variety of human diseases that result from overactive inflammatory responses [16].

Pathological pancreatic islet β-cell inflammatory responses are likely to be contributors to reductions in total numbers of islet β-cells as well as to reductions in insulin secretion that lead to either T1D or T2D [17,18,19,20,21]. Thus, strategies to limit or reduce pathological inflammation within β-cells may improve β-cell function and restore, or prevent losses in, β-cell mass. One such strategy is the use of botanical agents that have anti-inflammatory activity [22,23]. An example of a plant with a long history of use in human health and disease is *Artemisia dracunculus* L. (aka Tarragon). Extracts from *Artemisia dracunculus* L. have been reported to have a wide variety of beneficial properties, including, but not limited to, antibacterial, antifungal, anti-inflammatory, antioxidant, and antitumor activities [24]. Importantly, the oils and extracts from *Artemisia dracunculus* L. are deemed safe by the United States Food and Drug Administration [24].

Herein, we describe the use of an established and well-characterized ethanolic extract of *Artemisia dracunculus* L., designated PMI 5011, and compare it with 2′,4′-dihydroxy-4-methoxydihydrochalcone (DMC2), a compound purified from such extracts [25]. PMI 5011 has been documented to decrease NF-κB signaling in skeletal muscle [26] but this effect to suppress NF-κB activity has not been tested in pancreatic β-cells. We report that PMI 5011 and DMC2 inhibit p38 MAPK activity, reduce NF-κB signaling in β-cells, and restrict the transcription of genes that produce proteins responsible for immune cell recruitment. Collectively, these data support the concept that botanical-based approaches to downregulate inflammation are likely to be useful as direct or adjuvant therapies in a variety of human diseases. Understanding the anti-inflammatory nature of such botanical extracts and isolated compounds may provide novel therapeutics or adjuvants for the treatment of various human conditions with inflammation-associated causes.

## 2. Materials and Methods

### 2.1. Cell Culture, Islet Isolation and Dispersal, and Reagents

The establishment of 832/13 rat insulinoma cells has been previously described [27]. This cell line was maintained in RPMI-1640 (ThermoFisher Scientific, Waltham, MA, USA) with 10% fetal bovine serum (FBS) (ThermoFisher Scientific) and was determined to be free of mycoplasma contamination (MycoAlert Mycoplasma Detection Kit, Lonza, Basel, Switzerland). Islets were isolated from 4-month-old male C57BL/6J mice (Stock #000664, The Jackson Laboratory, Bar Harbor, ME, USA) using our previously published protocol (BBA PMID 25882704). Twenty-four hours after isolation, islets were strained using a 70 μm filter, handpicked, and dispersed. Culture media was removed, and batches of 100 islets were treated with 100 μL TrypLE (ThermoFisher Scientific) for 10 min at 37 °C. Then, 900 μL of media was added to quench the TrypLE, and dispersed islets were then seeded into wells of a 24-well plate. Once cells adhered, they were treated as shown in the respective figure legends using media without FBS. IL-1β was purchased from Peprotech (Cranbury, NJ, USA) and DMSO was purchased from Tocris Bioscience (Ellisville, MO, USA). Recombinant adenoviruses expressing 5x NF-κB-luciferase [28] and IKKβ S177E/S181E [29] have been previously described. CCL2 [30], CCL20 [31], and iNOS [32] promoter-luciferase constructs have all been previously described. 

### 2.2. Plant Material and Isolated Natural Product

An ethanolic extract of *Artemisia dracunculus* L. previously described and termed PMI 5011, as well as a fractional component of PMI5011 known as DMC2 (2′,4′-dihydroxy-4-methoxydihydrochalcone), which has been previously described [25,33], was supplied by the Botanical and Dietary Supplement Research Center at Pennington Biomedical Research Center. Phytochemical screening and the chemical structure of DMC2 have been documented previously [25].

### 2.3. Chemokine ELISAs

The detection of chemokines CCL2 and CCL20 secreted into the culture media was performed using ELISA kits from Abcam (Cambridge, MA, USA) according to the manufacturer’s suggested protocol. Chemokine release into the media was normalized to total protein to account for any potential differences in cell number.

### 2.4. Protein Isolation and Immunoblotting

Whole-cell lysates were prepared using M-PER (ThermoFisher Scientific). The isolation of nuclear and cytosolic extracts from 832/13 cells was performed using the ThermoFisher Scientific NE-PER nuclear and cytosolic extraction kit as directed by the manufacturer’s protocol. Cellular extracts were supplemented with protease and phosphatase inhibitor cocktails (Thermo Fisher Scientific), and the protein concentration was quantified by bicinchoninic acid (BCA) assay (ThermoFisher Scientific). The whole-cell lysate was run using SDS-PAGE, transferred to a PVDF membrane, and membranes were blocked prior to antibody incubation and subsequent downstream detection as described previously [34,35]. Antibodies used were the following: Anti-p65 (#8242), anti-PO4-p65 (#3033), anti-IKKβ (#8943), anti-IκBα (#4814), anti-PO4-IκBα (#2859), anti-PO4-p38 (#9126), anti-p38 (#8690), and anti-β-Actin (#8457) were all from Cell Signaling Technology (Danvers, MA, USA), while anti-iNOS (#160862) was from Cayman Chemicals (Ann Arbor, MI, USA). All primary antibodies were used at the manufacturers’ recommended dilution for immunoblotting in 1% PVP in TBS. HRP-linked anti-rabbit and anti-mouse IgGs were from Cell sinaling Technology (Cat #7074 and 7076, respectively) and used at a concentration of 1:5000. All chemiluminescent images were captured using a ChemiDoc Imaging System (Bio-Rad, Hercules, CA, USA).

### 2.5. Isolation of RNA, cDNA Synthesis, and Real-Time RT-PCR

Total RNA was isolated from cells and dispersed islets using TRI Reagent (Millipore Sigma, St. Louis, MO, USA). The synthesis of cDNA and real-time RT-PCR was performed using SYBR Green (BioRad Laboratories, Hercules, CA, USA) as previously described [28]. Primers used to detect transcript levels via RT-PCR were designed using Primer3Plus software and are available upon request.

### 2.6. Plasmid and siRNA Transfection, and Luciferase Assays

Transient transfections and/or the transduction of plasmids and siRNA duplexes into 832/13 cells and cell lysis for luciferase assays were previously described [36]. Briefly, transient transfection of plasmid vectors into 832/13 cells was performed using TransFectin Lipid Reagent (BioRad Laboratories, Hercules, CA, USA) according to the manufacturer’s instructions. Cells were then lysed in 1× Passive Lysis Buffer (Promega, Madison, WI, USA) and luciferase activity was measured using the Promega Luciferase Assay System in a plate reading luminometer (Promega). Luciferase activity was normalized to total protein content as determined by the BCA assay. 

### 2.7. Adenylate Kinase and MTS Assays

As an indicator of cell death, adenylate kinase released into the media was measured using the Lonza ToxiLight Bioassay according to the manufacturer’s protocol. MTS was used as a secondary analysis of cellular viability using the Promega CellTiter 96 Aqueous Cell assay. 

### 2.8. Glucose-Stimulated Insulin Secretion

First, 832/13 cells were grown in 12-well plates and treated as indicated. Glucose-stimulated insulin secretion (GSIS) assays were performed as described previously [27]. Secretion of insulin into the media was measured using the High Range Rat Insulin ELISA kit (Mercodia, Uppsala, Sweden) according to the company directions. Cells were lysed using an M-PER lysis reagent to quantify the total intracellular protein content via BCA assay. Insulin secretion data were normalized to total protein content to account for any differences in cell numbers between treatment groups. 

### 2.9. Statistical Analysis

One-way ANOVA analysis with Tukey’s post-hoc correction was performed using GraphPad Prism version 9.3.1. 

## 3. Results

### 3.1. PMI 5011 and DMC2 Do Not Promote β-Cell Toxicity

**Figure 1:**
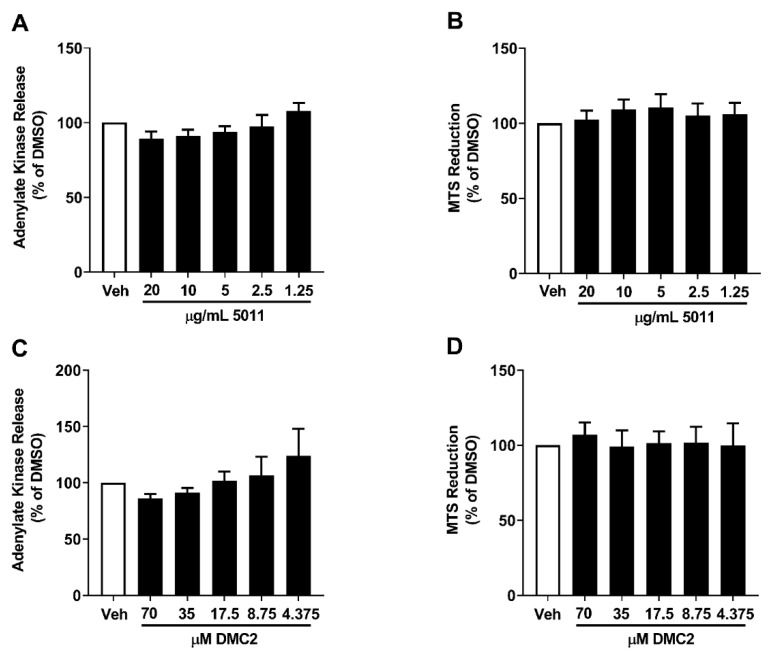
PMI 5011 (5011) and DMC2 do not promote β-cell toxicity. 832/13 cells were treated for 6 h with DMSO vehicle (Veh) or the indicated concentrations of both PMI 5011 (**A**,**B**) and DMC2 (**C**,**D**). Adenylate kinase release into the media (**A**,**C**) and MTS reduction (**B**,**D**) were determined. Data are shown as means ± SEM from 6 individual experiments.

An established and well-characterized ethanolic extract of *Artemisia dracunculus* L., designated PMI 5011, improves insulin action in vitro and in vivo [33,37,38]. As part of these effects, there is evidence that the downregulation of inflammation is likely a contributing factor [26]. Because inflammation is also associated with islet β-cell communication with immune cells, which influences autoimmunity [3,17], we tested the hypothesis that PMI 5011 and DMC2, an isolated compound from the PMI 5011 extract, could modulate sensitivity to IL-1β. First, we exposed the 832/13 rat insulinoma cell line to descending concentrations of PMI 5011 or DMC2. We found that 6 h of exposure to PMI 5011 did not increase adenylate kinase (ADK) release (Figure 1A), a marker of cell lysis, and did not alter MTS reduction, a marker of impaired mitochondrial function (Figure 1B). Similarly, DMC2 did not display toxicity in the ADK (Figure 1C) or MTS (Figure 1D) assays.

### 3.2. PMI 5011 and DMC2 Decrease IL-1β-Mediated NF-κB Activity

**Figure 2:**
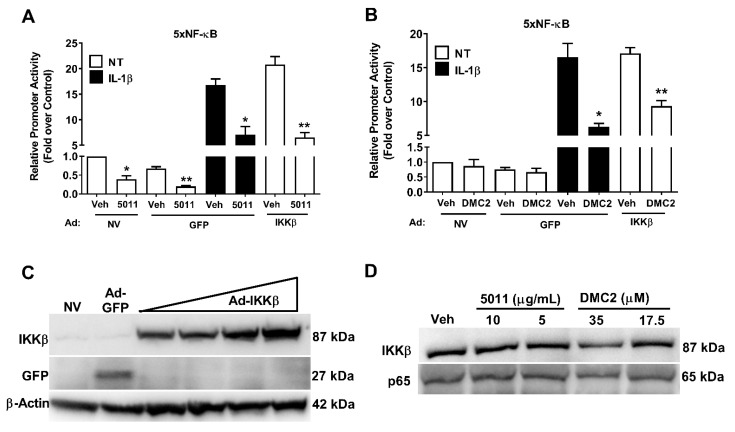
PMI 5011 and DMC2 decrease IL-1β-mediated NF-κB activity. (**A**,**B**) 832/13 cells were either untreated (NV; no virus) or transduced with adenoviruses overexpression GFP or constitutively active IKKβ overnight. The cells were exposed to PMI 5011 (**A**) or DMC2 (**B**) for one hour, then either left untreated (white bars) or exposed to 1 ng/mL IL-1β for 4 h (black bars). Luciferase activity was measured as a readout of NF-κB signaling. (**C**) 832/13 cells were transduced with increasing concentrations of an adenovirus that expresses constitutively active IKKβ or a single dose of an adenovirus expressing green fluorescent protein (GFP). 24 h post-transduction, cells were lysed, and total protein was assayed by Western blot for each of the indicated proteins. β-actin is shown as a control for protein loading. (**D**) 832/13 cells were exposed to the indicated concentrations of either PMI 5011 or DMC2 for one hour, then lysed for Western blot analysis of endogenous IKKβ and p65. *, *p* < 0.05; **, *p* < 0.01.

Next, we exposed the 832/13 rat insulinoma cell line to either IL-1β or IL-1β after one pre-treatment in the presence of either PMI 5011 or DMC2. We found that NF-κB activity was reduced in the presence of PMI 5011 in a basal state (Figure 2A; four white bars on left-hand side) and in response to stimulation with the pro-inflammatory cytokine IL-1β (Figure 2A; black bars). In addition, NF-κB activity driven by the overexpression of constitutively active IKKβ (caIKKβ) was also diminished by pre-treatment with PMI 5011 (Figure 2A; white bars right-hand side). Moreover, one-hour pre-treatment with DMC2 reduced NF-κB activity driven by IL-1β (Figure 2B). The overexpression of caIKKβ and the GFP control is shown in Figure 2C. We further found that the one-hour exposure of 832/13 rat β-cells to two concentrations of either PMI 5011 or DMC2 did not reduce the abundance of either IKKβ or p65 (Figure 2D). Taken together, these data are consistent with PMI 5011 and DMC2 reducing NF-κB activity by a mechanism other than simply decreasing IKKβ or p65 protein abundance.

### 3.3. DMC2 Reduces p38 MAPK Phosphorylation but Not Phosphorylation of Either IκBα or p65

**Figure 3:**
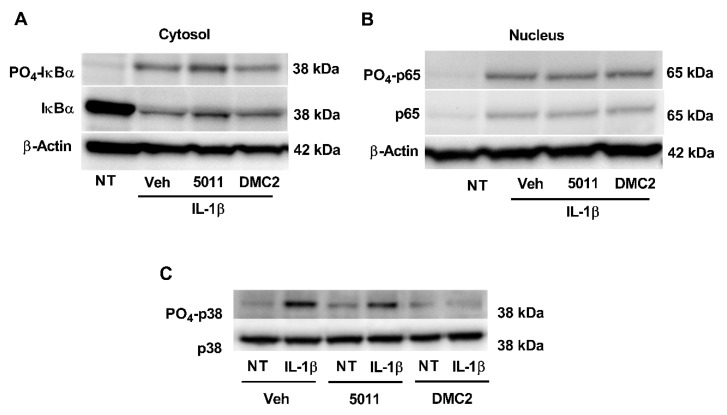
DMC2 reduces p38 MAPK phosphorylation but not phosphorylation of either IκBα or p65. (**A**–**C**) 832/13 cells were treated with DMSO (Veh), PMI 5011, or DMC2 for one hour, followed by exposure to IL-1β for 15 min. (**A**) Western blots for phospho-IκBα (S32), total IκBα, and β-actin (loading control). (**B**) Western blots indicating phosphorylation of p65 (S536), total p65, and β-actin. (**C**) Western blots showing phosphorylation of p38 MAPK (Thr180/Tyr182) and total p38 MAPK.

We next examined the degradation of IκBα, which restricts p65 nuclear translocation by holding p65 in the cytoplasm under basal conditions. A signal, such as IL-1R activation, promotes the phosphorylation and ubiquitin-mediated degradation of IκBα, allowing p65 nuclear translocation [39]. We found that the phosphorylation and degradation of IκBα in response to IL-1β were not altered by pretreatment with either PMI 5011 or DMC2 (Figure 3A). Similarly, neither phosphorylation nor nuclear translocation of p65 was changed by the presence of PMI5011 or DMC2 (Figure 3B). However, we observed that both PMI 5011 modestly and DMC2 strongly inhibited the phosphorylation of the p38 MAPK (Figure 3C).

### 3.4. PMI 5011 and DMC2 Reduce Ccl2 Gene Expression and Protein Secretion

**Figure 4:**
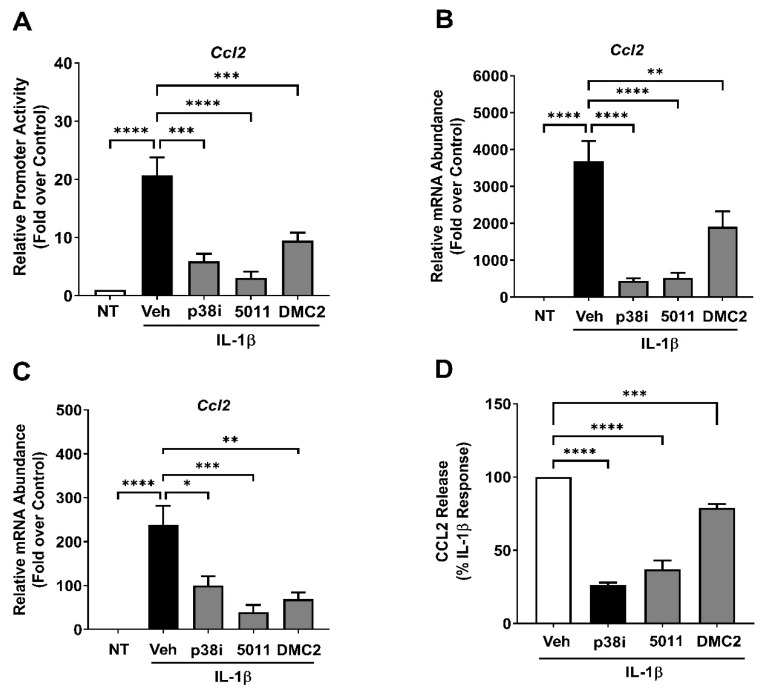
PMI 5011 and DMC2 reduce *Ccl2* gene expression and protein secretion. (**A**) 832/13 cells were transfected with a plasmid containing the −3.6kb *Ccl2* promoter driving a luciferase reporter. 24 h after transfected, the cells were treated with 10 μM of the p38 MAPK inhibitor SB202190 (p38i), 10 μg/mL PMI5011, or 35 μM DMC2 for one hour, followed by exposure to 1 ng/mL IL-1β for 4 h. Luciferase activity is plotted as the fold response over untreated (no IL-1β) veh control. (**B**) 832/13 cells were treated with 10 μM of the p38 MAPK inhibitor SB202190, 10 μg/mL PMI5011, or 35 μM DMC2 for one hour, followed by exposure to 1 ng/mL IL-1β for 3 h. Expression of the *Ccl2* transcript was measured by qPCR. (**C**) Isolated mouse islets were treated with 10 μM p38i, μg/mL PMI5011, or 35 μM DMC2 for one hour, followed by exposure to 1 ng/mL IL-1β for 3 h. Expression of the Ccl2 gene was analyzed by qPCR. (**D**) 832/13 cells were treated with 10 μM of the p38 MAPK inhibitor SB202190, 10 μg/mL PMI5011, or 35 μM DMC2 for one hour, followed by exposure to 1 ng/mL IL-1β for 6 h. Secretion of Ccl2 into the cultured media was assayed by ELISA. Data shown are expressed as the percentage of the maximal secretion response induced by IL-1β. *, *p* < 0.05; **, *p* < 0.01; ***, *p* < 0.001; ****, *p* < 0.0001.

Because PMI 5011 and DMC2 demonstrated the ability to reduce p38 MAPK phosphorylation in response to IL-1β, we tested whether they could reduce the expression of the *Ccl2* and *Ccl20* genes. We choose these two genes for their known sensitivity to NF-κB activity [29,31]. Again, one-hour pre-treatment with an established p38 MAPK inhibitor (SB202190), PMI 5011, or DMC2, suppressed the -3.6kb region of the *Ccl2* gene promoter by 71.4%, 85.2%, and 54.1%, respectively (Figure 4A). In addition, all of these treatments significantly restricted the IL-1β-mediated induction of the *Ccl2* gene in 832/13 rat β-cells (Figure 4B) and in isolated mouse islets (Figure 4C). The secretion of CCL2 protein was also blunted by 73.6%, 63%, and 21.3% in the presence of SB202190, PMI5011, and DMC2, respectively (Figure 4D). 

### 3.5. PMI 5011 and DMC2 Reduce Ccl20 Gene Expression and Protein Secretion

**Figure 5:**
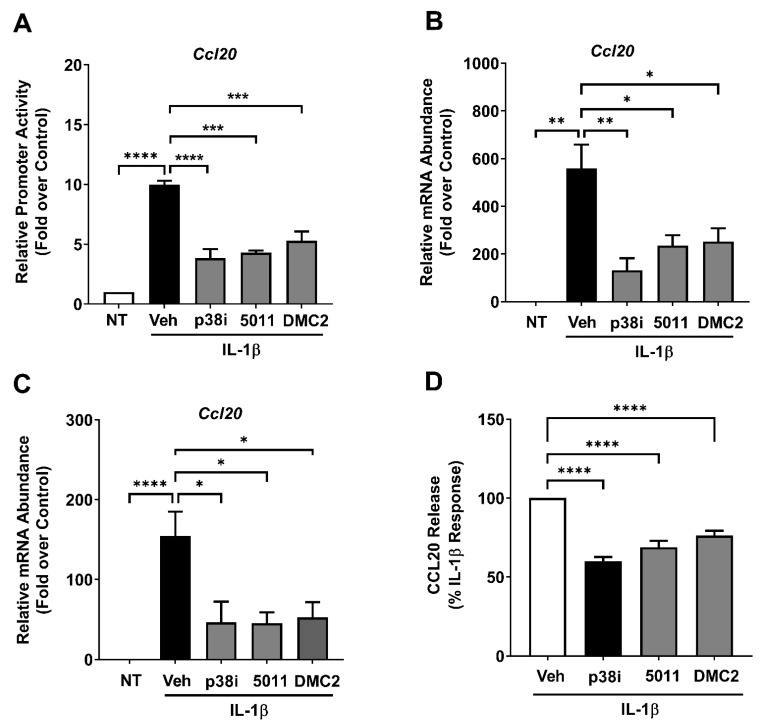
PMI 5011 and DMC2 reduce *Ccl20* gene expression and protein secretion. (**A**) 832/13 cells were transfected with a plasmid containing the −3 kb *Ccl20* promoter driving a luciferase reporter. 24 h after being transfected, the cells were treated with 10 μM of the p38 MAPK inhibitor SB202190 (p38i), 10 μg/mL PMI5011, or 35 μM DMC2 for one hour, followed by exposure to 1 ng/mL IL-1β for 4 h. Luciferase activity is plotted as the fold response over untreated (no IL-1β) veh control. (**B**) 832/13 cells were treated with 10 μM of the p38 MAPK inhibitor SB202190, 10 μg/mL PMI5011, or 35 μM DMC2 for one hour, followed by exposure to 1 ng/mL IL-1β for 3 h. Expression of the *Ccl20* transcript was measured by qPCR. (**C**) Isolated mouse islets were treated with 10 μM p38i, 10 μg/mL PMI5011, or 35 μM DMC2 for one hour, followed by exposure to 1 ng/mL IL-1β for 3 h. Expression of the *Ccl20* gene was analyzed by qPCR. (**D**) 832/13 cells were treated with 10 μM of the p38 MAPK inhibitor SB202190, 10 μg/mL PMI5011, or 35 μM DMC2 for one hour, followed by exposure to 1 ng/mL IL-1β for 6 h. Secretion of Ccl20 into the cultured media was assayed by ELISA. Data shown are expressed as the percentage of the maximal secretion response induced by IL-1β. *, *p* < 0.05; **, *p* < 0.01; ***, *p* < 0.001; ****, *p* < 0.0001.

We next examined how these interventions impacted the *Ccl20* gene in response to IL-1β as a separate validated NF-κB target gene [31]. We again observed that the p38 inhibitor SB202190 repressed the ability of IL-1β to activate −3 kb of the *Ccl20* promoter (Figure 5A) and induce expression of the *Ccl20* gene in 832/13 cells (Figure 5B). PMI 5011 and DMC2 also prevented the IL-1β-mediated transcriptional activation of the *Ccl20* promoter-luciferase vector (Figure 5A), as well as the induction of this gene in both cell lines (Figure 5B) and mouse islets (Figure 5C). Furthermore, all three treatments decreased the IL-1β-dependent secretion of CCL20 from cell lines (Figure 5D). 

Similar impairment of the IL-1β-mediated activation of promoter activity and gene expression of *Nos2*, another p38 MAPK-sensitive gene, are observed in the presence of PMI 5011 and DMC2 (data not shown). In addition, the protein abundance of iNOS (encoded by *Nos2*) was also significantly blunted (data not shown). In contrast, the incubation of 832/13 cells with PMI 5011 and DMC2 did not alter the expression of a number of other genes (e.g., *Cxcl1, Cxcl2*, etc.) that are not sensitive to inhibition by p38 MAPK (data not shown). Cumulatively, PMI 5011 and DMC2 each displayed inhibitory activity towards the p38 MAPK and reduced NF-κB signaling, indicating strong anti-inflammatory potential.

### 3.6. DMC2 Does Not Impair Glucose-Stimulated Insulin Secretion

**Figure 6:**
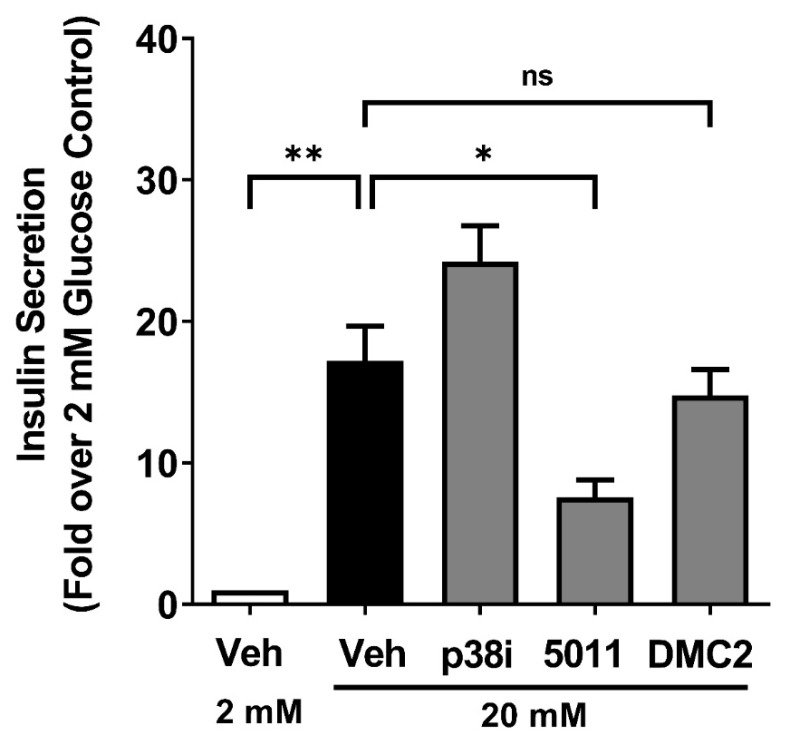
DMC2 does not impair glucose-stimulated insulin secretion. 832/13 cells were treated with 10 μM of the p38 MAPK inhibitor SB202190, 10 μg/mL PMI5011, or 35 μM DMC2 for one hour. Insulin secretion output after two hours of exposure to 20 mM glucose was measured by ELISA. The data shown as the fold response over the 2 mM (low glucose) control. *, *p* < 0.05; **, *p* < 0.01; n.s., not significant.

Next, we measured insulin secretion using the glucose-sensitive 832/13 rat β-cell line after exposure to either PMI 5011 or DMC2. We observed that PMI 5011 reduced glucose-stimulated insulin secretion by 56%, while SB202190 and DMC2 did not significantly alter insulin output (Figure 6). Collectively, it appears that the DMC2 fraction isolated from the ethanolic extract PMI 5011 has anti-inflammatory activity but does not impair insulin secretion.

## 4. Discussion

Medicines derived from plant sources are part of current clinical care [40,41]. Additional efforts to identify isolated compounds from botanical extracts have yielded new molecules that need further testing to fully identify their beneficial properties. In this study, we compared the well-characterized extract of *Artemisia dracunculus* L., termed PMI 5011, with DMC2, an isolated compound from that extract using the 832/13 rat insulinoma cell line and isolated mouse islets. Several key findings emerged from this approach: (1) PMI 5011 and DMC2 have anti-inflammatory effects. (2) These anti-inflammatory actions arise through the inhibition of p38 MAPK. (3) While PMI 5011 inhibits insulin secretion, DMC2 does not.

Many anti-inflammatory drugs currently prescribed were derived from plant-based sources [41,42]. One of the best-known examples is aspirin, where the salicylates from the willow leaves and bark have provided relief from fever and other ailments for centuries [42]. It was eventually discovered that the mechanism of action by which salicylates provided such relief arose from their ability to inhibit the cyclooxygenase enzymes [40]. Understanding the mechanism of action is a key component of the goal to refine and develop a novel therapeutic to limit diseases with inflammatory bases. As such, moving from plant-based extracts that show efficacy to identifying molecules with the desired action is a major goal. Towards this goal, the molecule DMC2 was identified from a fraction of PMI 5011 [25].

We have discovered that PMI 5011 and DMC2 reduce NF-κB signaling in pancreatic β-cells (Figure 2) and exhibit inhibitory activity against the p38 MAPK (Figure 3C). With the p38 MAPK family linked to diseases with inflammatory components and described as important targets to limit inflammation [43], this is an important first step to identifying new sources or molecules that function as inhibitors of this important signaling pathway. Importantly, we have identified that DMC2 retains its anti-inflammatory effects without impairing insulin secretion (Figure 6). DMC2 belongs to a larger class of molecules called chalcones, which include many bioactive molecules investigated for their preclinical and clinical benefits [44]. Interestingly, while DMC2 did not impair insulin secretion, exposure to PMI 5011 did reduce insulin output. We interpret these data to indicate that the ethanolic extract has additional components beyond DMC2 that are responsible for the reduction in insulin secretion observed in this study.

The chemical structure of chalcones, which includes DMC2, is a benzyl acetophenone. Several different types of plants contain the chalcone class of bioactive compounds and these flavonoids are recognized as beneficial in the treatment of various human diseases [45]. As a chemical class, chalcones have been reported to display anti-inflammatory activity, including the inhibition of cyclooxygenase and suppression of TNF-α derived NF-κB activity [46]. The chalcone DMC2 was isolated from a larger complex mixture identified from the ethanolic extract of *Artemisia dracunculus* L. (aka Taragon) [25]. We report herein the inhibition of p38 MAPK activity as part of the anti-inflammatory effects present within the overall extract that is retained by isolated, purified DMC2.

The use of p38 MAPK inhibitors to reduce immune cell invasion into pancreatic islets has been successful at the preclinical level [47]. Thus, botanical extracts containing p38 MAPK inhibitory, or unique low-molecular-weight p38 inhibitors, could potentially be conceived as direct or adjuvant therapeutics based on additional studies. Moreover, novel p38 MAPK inhibitors could have broader utility than just diseases with an autoimmune origin. For example, the inhibition of p38 MAPK may be able to limit COVID-19 infection or disease severity [48]. Thus, our discovery of a botanical-based compound (i.e., DMC2), which interferes with or directly inhibits p38 MAPK activity, could have broader impacts beyond reducing inflammation in pancreatic β-cells. 

In summary, we demonstrate herein that PMI 5011, an ethanolic extract of *Artemisia*, has potent anti-inflammatory activity in pancreatic β-cells. In addition, we have identified that a low-molecular-weight component contained within this extract, termed DMC2, has most, if not all, of the anti-inflammatory activity observed with the ethanolic extract. Specifically, DMC2 inhibits NF-κB and p38 MAPK activity and does so without impairing glucose-stimulated insulin secretion. 

## Data Availability

The data presented in this study are available upon reasonable request by contacting the corresponding author.
